# Anonymity-preserving Reputation Management System for health sector

**DOI:** 10.1371/journal.pone.0195021

**Published:** 2018-04-12

**Authors:** Farhana Jabeen, Zara Hamid, Wadood Abdul, Sanaa Ghouzali, Abid Khan, Saif Ur Rehman Malik, Mansoor Shaukat Khan, Sarfraz Nawaz

**Affiliations:** 1 Department of Computer Science, COMSATS Institute of Information Technology, Islamabad, Pakistan; 2 Department of Computer Engineering, College of Computer and Information Sciences, King Saud University, Riyadh, Saudi Arabia; 3 Department of Information Technology, College of Computer and Information Sciences, King Saud University, Riyadh, Saudi Arabia; 4 Department of Mathematics, COMSATS Institute of Information Technology, Islamabad, Pakistan; 5 Computer Laboratory, University of Cambridge, Cambridge, United Kingdom; Victoria University, AUSTRALIA

## Abstract

In health sector, trust is considered important because it indirectly influences the quality of health care through patient satisfaction, adherence and the continuity of its relationship with health care professionals and the promotion of accurate and timely diagnoses. One of the important requirements of TRSs in the health sector is rating secrecy, which mandates that the identification information about the service consumer should be kept secret to prevent any privacy violation. Anonymity and trust are two imperative objectives, and no significant explicit efforts have been made to achieve both of them at the same time. In this paper, we present a framework for solving the problem of reconciling trust with anonymity in the health sector. Our solution comprises Anonymous *Reputation Management* (ARM) protocol and *Context-aware Trustworthiness Assessment* (CTA) protocol. ARM protocol ensures that only those service consumers who received a service from a specific service provider provide a recommendation score anonymously with in the specified time limit. The CTA protocol computes the reputation of a user as a service provider and as a recommender. To determine the correctness of the proposed ARM protocol, formal modelling and verification are performed using High Level Petri Nets (HLPN) and Z3 Solver. Our simulation results verify the accuracy of the proposed context-aware trust assessment scheme.

## 1 Introduction

Trust is a multidimensional concept that is essentially relational or interpersonal and context-specific in nature and is largely dependent on the characteristics of both the trustor and the trustee within a specific relationship that varies in depth and strength over time. Existing studies indicate that trust is considered by users as an indicator of care quality and a patient’s experience with health services and is correlated with patient satisfaction [1]. According to a survey [2], trust facilitates commitment to organization, and advancements in collaborations between health care organizations.

Trust mechanisms can be divided into two categories: (i) soft (evaluation), and (ii) hard (authorization). Soft trust relationships are based on non-cryptographic mechanisms while the hard trust relationships are based on cryptographic mechanisms. Soft trust is context-dependent and is derived using individual or social control mechanisms. In the health sector, trust is continuously built upon the quality and the reliability of the HSP over time, i.e., by aggregating personal experiences **[3][4]**. However, in situations where the user inquiring trust does not have any personal experience with an HSP, to assess the trustworthiness of the HSP, he/she often asks other trustworthy peers about their experiences with the specific HSP (domain trust) or they ask for referrals to other trustworthy peers that have knowledge/experiences with the specific HSP (referral trust) **[4][5]**. Trust yields to evidence-based trust management, where the trust degree (reputation) is explicitly computed by a *Trust and Reputation System* (TRS) **[4][5]**. These systems help the service consumers make better, informed decisions regarding the selection of services/service providers that can be relied upon without the risk of damages from poor quality or even deceptive services. The trust degree represents the opinion of patients about health care professional and based on their evaluation their willingness to recommend health care professional **[6][7][8]**. In a survey **[9]** conducted in 2014, most users (82 percent) believed that an average rating of four stars or better was ‘good enough’ in the selection of a doctor. The top physician rating websites include RateMDs, HealthGrades, ZocDoc, and Vitals **[6][7]**. Similar to other service-oriented businesses, these doctor rating websites allow service consumers to evaluate their experiences and satisfaction with healthcare professionals or organizations **[6][7][8]**. Tara Lagu **[7]** suggested that rather than encouraging commercial websites to publish online information (ratings and reviews) about doctors and hospitals, hospital health systems should collect information about the quality of service received. Healthcare laws in most countries (such as the UK and Germany) require the collection of ratings and reviews based on patient experience, which can later be posted on doctors’ profile pages. In 2007, the UK government indicated its support for healthcare provider ratings by allowing the National Health Service (NHS) to launch the NHS Choices website, allowing patients to assess both healthcare professionals and organizations **[10].**

E-health exploits electronic processes and communication technologies to obtain, share or store information related to healthcare with the objective of improving healthcare services **[11]**. The main application areas of e-Health include Electronic Health Records (EHR’s) **[11, 12, 13]**, ubiquitous and pervasive health,‬ telemedicine & telecare services, and decision support system. EHR can be created, and managed by authorized users across more than one HSP. To use the potential advantages of e-health in a meaningful way, it is important to establish and manage trust among the entities participating in the whole process.

The nature of the Internet and of the healthcare domain presents new vulnerabilities because users may provide unreliable or malicious reports, reducing the reputation rating of service providers unfairly **[12][13] [14]**. The TRS should be able to identify and punish dishonest entities who attempt to bias trust values. To compute trust in the health sector, one of the important requirements is rating secrecy; that is, service consumer identity information is kept secret to avoid retaliation and privacy violations. Revenge rating is an issue especially in the health sector, where a service provider (e.g., a doctor), by acting as service consumer (e.g., a patient), may take revenge by spoiling the reputation of another service provider. For example, a cardiac physician may give bad ratings to an oncologist as a service consumer, who upon knowing may spoil the reputation of that specific cardiac physician as a service consumer. Bad-mouthing and negative discrimination can be avoided if the TRS allows the service consumers to provide ratings anonymously. Moreover, service consumers can act maliciously by providing false or misleading information. Therefore, anonymous online reviews/ratings can be particularly harmful to a physician’s reputation. Only the users having an interaction with a service provider should be allowed to provide ratings. It is important that service consumers are accountable for the feedback they provide about other service providers. Any malicious user trying to manipulate trust ratings should be identifiable. The TRS should know the identity of all service consumers and service providers and keep their identities concealed from one another **[12]**. In addition, it should record all dealings, ratings and the estimated reputations of service consumers and service providers. Service consumers must be able to provide ratings anonymously, i.e., their actions must be disconnected from their real-world identities and their other actions that can lead to the disclosure of their identities. However, anonymizing a user identity does not solve the problem. Anonymous ratings by one service consumer can be linked to each other since the ratings provided by a service consumer share the same pseudonym/identifier. This enables the system to easily build a profile of that individual. Individuals can be re-identified by exploiting real-world-related information about the individual (collected through external sources) along with data mining techniques. A daunting challenge in such systems is achieving both trust and anonymity.

There have been many research efforts that have investigated approaches to providing privacy in the e-health domain using hard trust (i.e., cryptographic) techniques **[11]**. However, providing anonymity while rating the quality of service received from the service provider in the health sector domain has not been considered. Currently, the existing work related to soft trust in e-health focuses on the information content dimension measuring the trustworthiness of the medical data held in EHR) [13][14]. In this paper, we present framework for an anonymity-preserving reputation management system in the health sector that focuses on service consumers and service providers (trustee) behaviours. The major contributions of this article are as follows:

- To prevent uncontrolled ratings that can be used to gain an unfair reputation, our work ensures that only those service consumers who received a service from a specific service provider can provide trustworthiness scores with in specific time period.
- An *Anonymous Reputation Management* (ARM) mechanism is proposed. The main contributions of the ARM protocol include: (i) avoiding linking of anonymous ratings provided by a service consumer, (ii) keeping the identities of service consumers concealed from service providers, and (iii) unveiling service consumer’s and service provider’s identities if required by the system.
- A context-aware trust assessment algorithm is presented to compute the reputation of entities as service providers and as recommendation source.
- Our simulation results verify the accuracy of the proposed CTA scheme. Moreover, through formal verification, we verify the ARM protocol.

The rest of this article is organized as follows: Section 2 reviews the related literature. Section 3 presents a privacy-preserving reputation management scheme, describing both the anonymization scheme and the reputation computation scheme. Section 4 presents formal modelling and verification using *High Level Petri Nets* (HLPN) and Z3 Solver of the anonymity preserving scheme. Section 5 presents the security analysis of the proposed anonymity-preserving scheme and the reputation assessment scheme and experimental evaluation of the reputation assessment scheme. Section 6 concludes the paper.

## 2. Related work

Currently, little work related to the TRS in the e-health sector exists. Therefore, this section describes the potentially competing research in the e-health domain rather than the literature related to solving the identified challenges in any other related application area.

### 2.1 E-Health domain

There exist some investigations that addresses the requirement for the computation of HCP reputations, but either their solution is not complete or is, in some cases, too basic [3–9]. Moreover, most of such research results focus on the fundamental characteristics of physician rating sites, including the frequency, the content, quality measures, and user assessment patterns. To date, direct evidence at physician rating sites regarding the following is lacking: (i) techniques and trust models that have been incorporated to compute the reputation of HCPs, (ii) discussion regarding the requirements of the TRS that were and were not met in the healthcare domain, (iii) the robustness against attacks expected on the TRSs in healthcare, and (iv) the incorporated privacy and security techniques and trust model. Privacy-preserving techniques based on cryptographic techniques have been thoroughly investigated in the context of e-health **[11]**. Commonly used disclosure control techniques include de-identification **[15]**, anonymization **[16]** and pseudonymization **[17]**. Some works have surveyed the existing privacy-preserving techniques to investigate how access is granted to individual health records while maintaining patient’s privacy.

Hedaquin [14] is a system that allows to evaluate the quality of the healthcare data in a patient’s health record. A reputation engine calculates a reputation using local, global, rule and aggregation ratings. Rule ratings are collected from a rule engine that assigns ratings to service providers on the basis of his/her degree, certificates and practices. Aggregation ratings are collected from an aggregation engine that performs a comparison of measurements from two data health suppliers on the same person. If the measurements are the same, then the reputations of both supplier’s increase. Otherwise, they decrease. Hedaquin [14] assigns the constant value (0.5) to the newcomers.

Alhaqabani et.al. [13] propose a model to measure the trustworthiness of medical data stored in the patient’s EHR. Similar to our work, Alhaqabani et. al. [13] and Deursen et. al. [14] assume the existence of a health authority that records information related to medical negligence. For a specific HSP, the rating is computed based on information received from the health authority, recent reputation scores and reputation score history (collected by the reputed centre). Moreover, Alhaqbani et. al., [13] also presented a pseudo anonymity technique in which a unique local ID is assigned to each patient at each individual HSP. The patient responsible for linking the medical records stored at different HSP’s. To allow records aggregation from different HSP’s, the patient is required to link himself/herself to a specific HSP (using the pseudonym provided by the HSP) as long as the HSP is in a trust agreement with the TTP. Pseudonyms are used to preserve patient anonymity and are used as a reference when an HSP wants to exchange data about the patient. Our work focuses on providing anonymity to the user and computing the reputation score of a service provider, but they focused on measuring the quality of medical data. Jøsang et. al. [12] presented the research agenda for the development of robustness mechanisms for trust and reputation systems.

Ruotsalainen et al. [18] present a privacy-enabled architecture based on Trusted eHealth and eWelfare Space (THEWS) principles. The THEWS principles focus on the rights to be given to the data subject to control the access and use of personal information in ubiquitous healthcare. The presented architecture makes use of existing work related to soft trust for trust verification, policy management and context-awareness in the construction of privacy policies. To allow secondary use of healthcare information, the concerned individual is allowed to set context-aware personal privacy and trust. To improve the privacy and security of data communication in Wireless Body Area Network (WBAN), the trustworthiness requirement among users is significant. In [4], a trust based scheme is presented for reliable and trustworthy collection of patient's physiological data. To evaluate the telemedicine based cloud computing services, Waqar et al. [3] presented cloud reputation evaluation model which consider the quality of services provided.

### 2.2 Other domains

The unlinkability of service consumer actions in different contexts can be attained by context-specific pseudonyms **[19]**. Androulaki et al. **[20]** use transaction pseudonyms to avoid linkability between transactions. In the case of a distributed reputation management system, to protect the privacy of those who consult peers to acquire the reputation of a service provider, Pavlov et al. proposed the use of anonymous communication **[21]**. Resnick et. al. **[22]** stated that the use of disposable pseudonyms results in cooperation hindrances in the absence of centralized domain. There is a high likelihood of misbehaviour if users are allowed to generate pseudonyms by themselves. The service consumer may authenticate himself with a pseudonym and misbehave, and after that he/she create a new pseudonym, and by using the new pseudonym misbehave again. To overcome this problem, the authors suggested using free but irreplaceable pseudonyms that is certified through a blind signature by a central authority. Seigneur and Jensen **[23]** presented concept of context-dependent pseudonyms to address the problem. However, their approach suffers from “Sybil attacks”. Tormo et. al., **[24]** presented a trust model that supports privacy-preservation. To aggregate ratings from service consumers about a specific provider in a privacy-preserving way, the model incorporated homomorphic encryption technique. A limitation of the presented scheme is that it restricts provision of customized reputation values to each entity. Gal-Oz et al. **[25]** also emphasized the applicability of Homomorphic encryption to trust and reputation management systems. To keep the recommendation provided by a service consumer private, the technique aggregates recommendations from service consumers in distributed systems. One of the limitations of this work is that no involved entity in the system is able to unveil the actual recommendation value or feedback. Therefore, the involved entities are unable to compare feedback to determine the similarity between two service consumers. In contrast, our scheme does allow the determination of the similarity between two service consumers. Christin et. al. **[26]** presented an anonymity scheme based on multiple pseudonyms for the participatory sensing system. This scheme allows the linking of interactions in a unique period while limiting the linking across multiple periods. In contrast, our scheme allows the linking of interactions across multiple periods. Advances in mobile technology coupled with the rapid advancement in the social networking lead to the concept of Pervasive Social Networking (PSN) [27]. In a social network, various content information flows. To identify the accuracy of public messages, in [27] a reputation-based messaging scheme is presented which uses group signatures and direct anonymous attestation. Group Signatures (GS) represent that the signer is a member of a group without revealing his/her identity. Direct Anonymous Attestation (DAA) allows to link messages with the same author and to link multiple feedbacks on a message. The proposed scheme requires the users to regularly update their secret keys used for DAA and GS.

In **[28]**, a trust model is proposed which incorporates a mechanism for assessing and adjusting the credibility of witnesses. It computes the fairness of witness ratings by comparing a witness's ratings with a service consumer's ratings provided to comparable (common) service providers. Witnesses' testimonies are computed based on the probability values obtained from assessing the fairness of witness ratings. The underlying mechanism is quite time-consuming because it requires the scanning of the witnesses' entire rating history for all provider’s every time while estimating the probability of witnesses' testimony fairness. To improve the accuracy of the transaction feedback, Singh et. al., presented a mechanism that requires proof of interaction (a certificate signed by both interacting parties) **[29]**. The proof of interaction will allow the computation of whether the two parties were actually involved in the transaction for which the rating is provided. This mechanism will prevent a malicious service consumer from providing dishonest feedback about service providers with whom they have not interacted. However, trusting a peer’s feedback and trusting a peer’s service quality are two different concepts.

The EigenTrust system **[30]** was developed to compute a reputation solely for the purpose of file exchange in the network. Normalization is applied to recommendations to discourage malicious peers from assigning high trust values. The normalized trust values do not distinguish users with negative reputations from users with neutral reputations. It does not consider the total number of interactions that a pair of peers had, allowing malicious users to manipulate the system at a low cost. The model assumes that service providers who are sufficiently trustworthy to provide a service are also sufficiently trustworthy to provide a recommendation. This assumption allows a malicious user to attack the trust and reputation system by providing high-quality service.

To address the unfair ratings problem, the authors proposed the use of collaborative filtering techniques **[31]** to group the recommenders whose ratings are in close proximity in a given context (called a similarity calculation). In **[32]**, after applying collaborative filtering, a divisive cluster-filtering algorithm is applied to separate ratings for a service provider into two clusters: one containing lower ratings and one containing higher ratings. Ratings in the higher rating cluster are considered unfair ratings and are therefore excluded. The limitation of the proposed approach is it cannot work efficiently with unfairly low ratings. FIRE **[33]** is a decentralized model that calculates a reputation based on the information collected from sources such as interaction trust, role-based trust, witness (third-party) reputation, and certified reputation. FIRE implements a witness reputation system that provides a form of referral reputation. It makes use of the confidence interval to compute the confidence of computed trust values. If the confidence is low, the peer gathers information about the service provider from the witnesses. It computes the fairness of witness ratings by comparing a witness’s ratings with a service consumer’s personal ratings regarding the commonly rated service provider. The underlying mechanism is quite time-consuming because it requires the scanning of the witnesses’ entire rating histories for all providers’ every time while estimating the probability of witnesses’ testimony fairness. This method assumes that users behave consistently, which may not be the case in camouflage attacks.

Malik and Bouguettaya **[34]** distinguish recommendations using rater credibility. The work assumes that only highly reputed recommenders can give honest recommendations. In **[35]**, a filtering algorithm is proposed that filters out the recommendations that does not lie between lower and upper quartiles. An assumption that is taken in to consideration by this technique is that recommendations follow beta distribution. This technique works effectively only if the data sets is large. Weng et al. in **[36]** proposed an entropy-based filtering mechanism. A recommendation is considered malicious if it appears too different from the majority opinion. Deno et al. **[37]** proposed an iterative filtering method. average of all received recommendations is calculated to reduce the impact of dishonest recommendations. In **[38]**, the authors used a control chart method to identify malicious recommendations. The control chart method calculates the lower and the upper confidence limits using the average and the standard deviation to filter out the recommendations that are malicious. In **[39]** scheme based on dissimilarity measure is presented to detect malicious recommendations. The scheme focuses on declaring the recommenders as malicious whose provided recommendation is distant from the *Median* value and has a lower frequency of occurrence. All the recommendation values are divided in to equal size buckets. Each bucket is represented with ID, i.e., largest recommendation value that can lie in it. The median is computed based on the buckets ID that are not empty.

## 3. Proposed reputation management protocol

In countries such as the UK, every citizen is registered with the *National Health Authority* (NHA). NHA assigns unique *National Health Service* (NHS) number to every requestor in order to avail medical services **[9]**. The NHS Number supports privacy because it supports de-identifying identification information from medical records. All the personal identification information is stored at the NHA and NHS number is provided to patient [40]. In our scheme, the *Health Care Authority* (HCA) provides the services of the NHA. Each user (service consumer and service provider) is assigned an NHS number by the national HCA to hide his or her identification information. For the accuracy of the TRS, only service consumers who received services from service providers can provide recommendations. For this purpose, a privacy-preserving framework is required that addresses the following: (i) preservation of patient privacy by hiding patient identification information, (ii) verification of the interaction information (i.e., the *Health Care Organization* (HCO) where the interaction occurred, the context, date and time when the interaction took place, and with which HSP), and (iii) anonymous time-bound pseudonym assignment (called a ticket) to the service consumer to provide a rating.

One solution to provide anonymity is to convert a patient's NHS number into a pseudonym (called the Global Identifier (GID)). The service consumer can enter a rating using the given pseudonym. An independent *Trusted Third Party* (TTP), such as the HCA, can perform such encoding. The user identity can be hidden using a pseudonym, but if a user maliciously acquires a user GID, the malicious user can easily link the anonymous ratings by one service consumer. Furthermore, generating an anonymous identity for each service consumer just for the purpose of submitting a rating is too costly. To justify this cost, we present a privacy-preserving architecture that supports the privacy-preserving TRS scheme and supports privacy-preservation in the distributed e-health system (each HCO is responsible for storing its own data). The *GID*_*i*_ of patient *i* can be used to store medical records at each HCO. However, if the same *GID*_*i*_ is stored against the healthcare tuples of patients in all healthcare organizations, then an internal attacker with a specific *GID*_*i*_ can obtain the healthcare tuples of a patient *i* with ease from different HCOs. To overcome such weakness, a unique *Local Identifier* (*LID*_*ij*_) is generated by the HSP *j* for each patient *i* based on his/her *GID*_*i*_. *LID*_*ij*_ of a patient support avoiding the possibility of linkage between medical data of i stored in different HCOs. *LID*_*ij*_ will be used by HCO *j* for the generation of the local medical records identifiers (*R*_*ijk*_) of a patient *i*. *R*_*ijk*_ hides the linkability between *LID*_*ij*_ and local medical records identifiers of patient *i*. Using the reversible pseudonym technique, *LID*_*ij*_ can be re-generated from the *R*_*ijk*_ when required to map records to the local pseudonym *LID*_*ij*_ of the patient in HCO j (the generation details of *R*_*ijk*_ are beyond the scope of this paper instead of [41]).

Another problem to address is to decide which entity should perform reputation management. If an HCO is responsible for authentication and reputation management, the service consumers could be easily traced through their dealings with the HCO, as their interaction information are maintained and coupled with their accounts at HCO. One of the solutions could be to delegate the reputation management functionality to the TTP that is considered to be a trustworthy party. Due to the privacy concern, still many service consumers may be unwilling to send their recommendations to the TTP. To overcome the privacy issues, the personal identification information of the users and the reputation scores they provided to the service providers should not be known by the same entity. Therefore, an independent *Reputation Management Centre* (RMC) should perform reputation management. To achieve unlinking ability in the reputation scores provided by the service consumer *i*, a unique pseudonym (i.e., tickets) can be assigned to the service consumer after every interaction with the service provider. This will be facilitated by the following: (i) service providers cannot trace which service consumer provided the rating, and (ii) similarity between the service consumers cannot be determined.

To achieve a common goal in multi-party computations, security constraints may be relaxed by assuming that different entities will not conspire **[42][43]**. In our proposed approach, the authentication process for all the service consumers who want to provide trustworthiness scores (ratings) based on the quality of service received from service providers is delegated to the HCO where they received the service. The HCO authenticates the users and verifies the interaction. The HCO provides an attribute certificate containing a unique time-bound token (called a ticket) and some other related information to the RMC. Therefore, the RMC can verify the authentication process, and service consumers can provide a rating without compromising their privacy.

To preserve the privacy of the service consumers, in our framework, we made efforts to fulfil the following privacy-related properties.

P.1 The TTP and the HCO must not know about the ratings given by service consumers.
P.2 The TTP and the HCO must not infer the relationship between two service consumers. They should not be able to determine similarity between two service consumers.
P.3 The RMC must not know the identification information of the service consumer that has provided a rating.
P.4 An internal attacker should not be able to identify whether two different interactions have been performed by the same service consumer at the RMC.
P.5 An internal attacker should not be able to link anonymous ratings provided by a service consumer at the RMC.
P.6 A data interpreter service as a secure mechanism at the RMC can extract the similarity between two service consumers and can correlate different service consumers’ interactions given their pseudonymized identifiers.
P.7 The TTP can determine the real identification information of the service consumer by converting the pseudonymized identifier (*GID*_*i*_) back into his/her NHS number. Once the NHS number is obtained, the Health Care Authority (HCA) can be contacted for the personal identification information of the entity bearing the NHS number.

### 3.1 Anonymous Reputation Management (ARM)

This section explains how service consumer Alice can anonymously rate service provider Bob after having an interaction with him in HCO j. To use medical facilities, as part of the process of registering with the HCA, the HCA will generate an NHS number and direct Alice’s request to the TTP by providing Alice’s NHS number.

#### 3.1.1 Generation of an anonymous GID by the TTP

**Fig 1:**
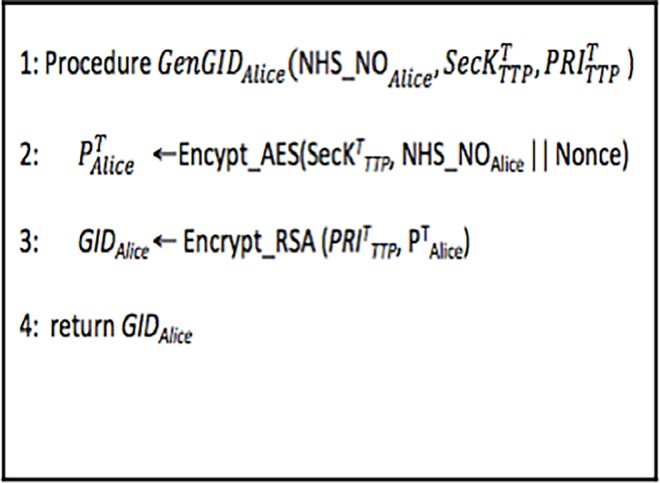
Algorithm 1: Generation of *GID*_*Alice*_.

As depicted in Fig 1, the patient Alice GID (*GID*_*Alice*_) is generated as follows. Firstly, *NHS_NO*_*Alice*_ is encrypted using the secret key (${SecK}_{TTP}^{T}$) of the TTP to generate her private key $P_{Alice}^{T}$. *Advanced Encryption Standard* (AES) algorithm **[44]** is used for encryption. Afterwards, the *GID*_*Alice*_ is generated by encrypting $P_{Alice}^{T}$ using TTP’s private key (${PRI}_{TTP}^{T}$) **[45]**.

To obtain *NHS*_*NO*_*Alice*_ from the ciphertext *GID*_*Alice*_ the decryption can be performed in two steps.

$$P_{Alice}^{T}=Decryption\;(PU_{TTP}^{T},\;GID_{Alice})$$

$$NHS\_NO_{Alice}\|Nonce=Decryption\;(SecK_{TTP}^{T},\;P_{Alice}^{T})$$

#### 3.1.2 Generation of an anonymous LID by the HCO

To interact with doctor *Bob* in HCO *j* that is registered with the HCA and TTP, patient *i* (i.e. Alice) first needs to register with the HCO by providing the identity certificate [46]. The HCO generates an *LID*_*Alicej*_ for patient Alice using symmetric key cryptography. The symmetric key of the *HCO*_*j*_ (i.e., ${SK}_{j}^{HSP}$) is encrypted using its secret key, ${SecK}_{j}^{HSP}$ and stored in the encrypted form to enforce security.

**Fig 2:**
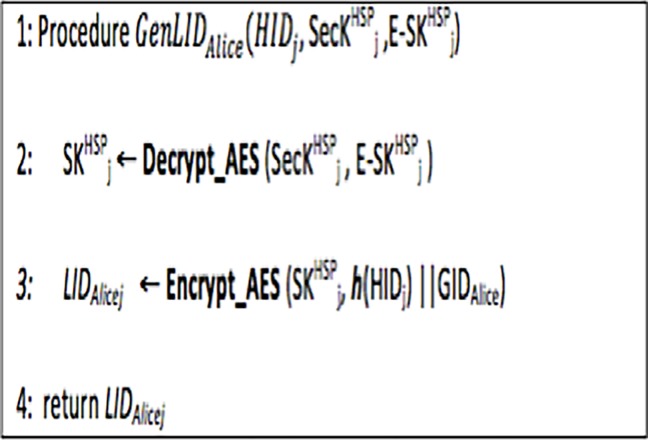
Algorithm 2: Generation of *LID*_*Alicej*_.

The HCA generates *HID*_*j*_ using the name and location of the hospital. The hash code *of HID*_*j*_ (**h**(*HID*_*j*_)) is computed using SHA-256 algorithm [47]. As depicted in Fig 2, the HSP uses its symmetric key (${SK}_{j}^{HSP}$) to encrypt the hash code of *HID*_*j*_ ***[47]*** and *GID*_*Alice*_ to generate the *LID*_*Alicej*_.

#### 3.1.3 Generation of the context identifier

In our reputation model, all the ratings for a service provider are not treated equally. Instead, a service consumer is required to rate an HSP in a specific context. The context includes the information regarding disease classification and doctor classification. For example, a service consumer might want to rate an HSP based on his interaction with that HSP after a surgical procedure, but another may rate an HSP based on an interaction in which the medical practitioner discussed his/her reports related to diabetes. Moreover, the overall reputation of the hospital should not decrease based on the bad reputation of one department. The diseases can be classified using different classification systems. A number of coding systems define a mechanism to code disease signs and symptoms, such as the *International Statistical Classification of Diseases and Related Health Problems* 10th Revision (ICD-10) **[48][49]**. The ICD-10 code presents a basic structure to code diseases defined as follows: Characters 1–3 define the particular category of a disease; the 4th character defines the aetiology of a disease; the 5th character defines the body part affected; the 6th character defines the severity of illness; and the 7th character is reserved for the extension of the code **[48]**. For example, G00-G99 represents Inflammatory diseases of the central nervous system, G10-G14 Systemic atrophies refers to the category of diseases of the nervous system **[49]**. Most of the countries, including UK and the US implemented the ICD-10 code. In our scheme, we assume that the HCA uses the ICD-10 coding scheme and that all the HCO’s are provided with the required information. Role refers to the role of the medical practitioner, whether he/she acted as a physician, a surgeon, a nurse, etc. In our work, the ***ContextID***_***c***_ contains the following information:

$$ContextID_{c}=Characters\;1–3\;(disease\;category)\;\|\;4\;\left(disease\;etiology\right)\;\|\;5\;\left(RoleID\right)$$

#### 3.1.4 Interaction with the HSP

**Fig 3:**
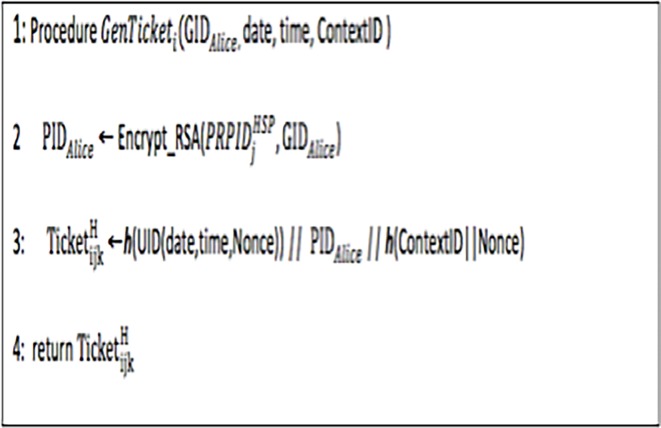
Algorithm 3: Generation of *Ticket*.

Each HSP (such as a nurse or a doctor) is assigned a unique ID called an HSPID. All HSPIDs and corresponding HSP names at HCO *j* are shared with the RMC so that the HSP reverses it to the HSP name before publishing reputation information. After an interaction with Bob, an interaction ID is assigned to the Alice, allowing her to enter a rating, and the required information about the interaction (such as the HSPID, the HID, the date of the interaction, the time of the interaction, the ContextID, etc.) will be stored in the local database. For each interaction k, unique ticket number Ticket_ijk_ is generated by the ticket-generation component at HCO *j* for patient Alice (depicted in Fig 3). At the time of ticket generation, the *LID*_*ij*_ of the service consumer in the interaction record is replaced by his/her *GID*_*i*_ information. The UID (date, time) returns a unique identifier based on the date and time of the interaction and a random nonce.

To encrypt the ticket, HCO j uses the private key *PRPID*_*j*_^*HSP*^. Each HCO shares the corresponding public key pair with the RMC. From Fig 3, it can be seen that ${Ticket}_{ijk}^{H}$ will be unique for every interaction between service consumer i and any service provider j. The service consumer can provide a rating of the provider’s performance within the specified validity period. The *data interpreter service* is a secure mechanism at the RMC that can extract the pseudonymized identifier (PID_*Alice*_) from ${Ticket}_{ijk}^{H}$ (by separating ***h***(UID(date, time, nonce)) and ***h***(ContextID_***c***_||Nonce) from ${Ticket}_{ijk}^{H}$). Therefore, the *data interpreter service* can compute the similarity between two service consumers and can correlate different service consumers’ interactions when required. Moreover, PID_*Alice*_ can be converted to GID_*Alice*_ when required to interlink the reputation records provided by Alice for different HSPs in different HCOs. If required, the TTP can be requested by the RMC to decrypt GID_*Alice*_ to obtain Alice’s NHS number. Moreover, the identification information of the user can be requested from the HCA using the NHS number.

#### 3.1.5 Authentication and reputation scores submission

Using the interaction ID, Alice can submit the reputation score. The submission process is as follows:

R.1 Alice accesses the web portal provided by the RMC to submit a reputation score.
R.2 The RMC redirects Alice to his/her HCO *H*_*1*_ *(where the interaction occurred)* with a request for authentication (defined using SAML **[50]** or OpenID**[51]**). In OpenID, service consumer is required to have a unique OpenID for each HCO.
R.3 *H*_*1*_ authenticates Alice and verifies her interaction with Bob at *H*_*1*_, represented by a specified interaction ID.
R.4 Once the HCO validates the required information about the specified interaction, the HCO redirects Alice to the RMC, with authentication statement. The authentication statement includes the generated attribute certificate.
R.5 After the validation of the authentication statement, RMC responds to the service consumer access requests, allowing/disallowing the consumer to enter a reputation score on the website.

Moreover, each HCO shares another asymmetric key pair with the RMC, private key (${PRSIG}_{j}^{HCP}$), which is used to generate an issuer signature and encrypt the attributes included in the attribute certificate. An attribute certificate **[52]** contains the following: $\left(i){Ticket}_{ijk}^{H},(\mathrm{ii}\right)$ Enc-RSA (${PRSIG}_{j}^{HCP}$, h(HSPID)||X||h(ContextID_***c***_)||X||UID-DT(date of the interaction, time of the interaction) ||X||validity period), (iii) a time stamp, (iv) the HID, and (v) issuer’s signature. X is used as a separator string to separate the attributes from the ciphertext after decryption. Symbol || represents concatenation. The issuer signature is generated by taking the hash of the contents of the certificate and by signing the hash value using the private key of the issuer. The UID-DT function returns a unique identifier that uniquely identifies the date and time of the interaction. The validity period of the certificate is set based on the date and time of the interaction. Let us consider an example scenario. If the RMC allows the service consumer to enter a reputation score within 15 days of his/her date of interaction with an HSP and the service consumer tries to enter the rating after 14 days, then the validity period of the certificate will be set as 1 day. The rating provided using ${Ticket}_{ijk}^{H}$ will be stored at the RMC with the attributes: ***h***(HSPID), ContextID_***c***_, UID-DT, HID (where the interaction occurred) and ${Ticket}_{ijk}^{H}$. The storage of these attributes at the RMC is required for a reputation assessment by the RMC as described in the next section.

### 3.2 Context-aware Trustworthiness Assessment (CTA) protocol

In our work, we assume the use of a component as proposed in **[**13] to compute the reputations score by the HCA. The HCA is responsible for assigning a base rate to medical practitioners/medical staff and hospitals that have freshly entered the healthcare division in the country. It computes the base rate based on experience in reputable health organizations and degrees acquired. To assign a base rate to hospitals/clinics, it considers the data quality, the logistics for the delivery of effective technical support, effective change processes, diagnoses and treatment quality, service availability, service accessibility, and possession of a well-trained workforce (having the required certifications and qualifications). HCA considers the reports received from the community regarding the HCO or the HSP. Moreover, in each hospital where the health service provider works, it collects timely information about his/her performance. The data related to HSP ability, benevolence, and integrity can be collected online through survey forms completed by the HSP and endorsed by the HCO. For example, for surgeons, the data collected includes the number of surgeries performed, the number of deaths, etc.

The RMC is responsible for the following three tasks. First, it computes the reputation score of the HSP using the CTA. Second, it collects the reputation score of the HSP from the HCA. Third, it computes the final reputation score based on the reputation score provided by the HCA and the CTA. It is also responsible for publishing the reputation scores.

After the passage of specified time period t, the reputation score is recalculated and updated. To calculate the final reputation (trustworthiness) score $FT(j,C_{x},t)$ (depicted in Eq 1) of service provider j, the weighted average is calculated based on the reputation scores provided by the HCA and the RMC. The trustworthiness value (reputation) of the service provider is calculated separately by the HCA ($T_{HCA}(j,t)$) and by the RMC ($T_{RMC}(j,C_{x},t)$).

$$FT\left(j,C_{x},t\right)=\frac{W_{\mathrm{HCA}}*T_{HCA}\left(j,t\right)+W_{\mathrm{RC}}*T_{RMC}\left(j,C_{x},t\right)}{W_{\mathrm{HCA}}+W_{\mathrm{RC}}}$$ (1)

#### 3.2.1 Reputation as calculated by the RMC

A malicious service consumer may initially provide fair interactions, with the intention of establishing credibility as a reliable recommendation source, only to deploy an attack once trust is gained. The malicious user may try to hurt the reputation of one or more good trustees by assigning unfairly low ratings to them. Therefore, the reputation model should incorporate a consumer’s past behaviour in the calculation of his/her reputation to accurately reflect his/her behavioural pattern. Furthermore, an HSP may act maliciously by providing high-quality services on high-demand trivial aspects and low-quality services on crucial aspects that are comparatively low in demand but associated with large financial gain. Moreover, HSPs may engage in fake interactions to artificially inflate their reputations and ratings. A trustworthy service provider may change his/her behaviour with the passage of time. The TRS can only be protected from malicious attempts if regulations with credible sanctioning options exist or technical mechanisms that can detect and discard malicious attempts.

Our scheme allows the service consumer to provide ratings on a 7-point rating scale rather than rating the quality of service provided by an HSP as true or false. In our scheme, the reputation computation engine at the RMC calculates the reputation score as follows.

#### 3.2.2 Incorporation of time-sensitivity aspect

The trustworthiness of the entity as a service provider and as a recommender is time dependent. To ensure the correctness of the computed reputation, it is necessary to consider the temporal behaviour of the concerned entity.

In many applications, such as stock market data, feedback (representing the quality of service received) in electronic communities, data may take the form of a stream of values. The services provided by each health service provider over a period of time results in the generation of a data stream of tuples (containing trustworthiness values) provided by the service consumers based on the quality of service received. In the proposed approach, the ratings regarding the Quality of Service (QoS) received by the service consumers are divided into two time windows: the recent time window *t*_*r*_ [53]**[54]** and the past time window *t*_*0*_. *t*_*r*_ stores the recent ratings received for service provider *j* in context C_x_. The recent time window dimension is in the form of FROM Start TO End, containing all the tuples (trustworthiness values based on the quality of service received from the service provider) that fall within a given range. The Start and End range of a window size is represented using time units (such as DAYS or MONTHS). Let *t* represent the time when time window t_r_ expires, upon which the service provider trustworthiness score is evaluated. Initially, the service provider is assigned a default reputation score **[14]**.

$T(j,C_{x},t_{r})$ represents the trustworthiness score and is computed based on the ratings received for service provider j, in context C_x_ in t_r_ (computed using Eq 6). Weighting of factor δ is done to give weight to the trustworthiness score computed at ***t***_**0**_ and t_r_ while updating the trustworthiness score of a service provider at t, as shown in Eq (7). Upon the expiration of t, before computing the updated trustworthiness value based on *t*_*r*_, $\left(i.e.,T(j,C_{x},t_{r})\right)$, the current value of $T(j,C_{x},t_{r})$ is stored in $T(j,C_{x},t_{r-1})$ and the trustworthiness score $T(j,C_{x},t_{0})$ is updated using Eq (2) to provide a time-sensitivity aspect.

$$T\left(j,C_{x},t_{0}\right)=\delta T\left(j,C_{x},t_{0}\right)+\left(1-\delta \right)T\left(j,C_{x},t_{r-1}\right)$$ (2)

Weighting factor δ is used to give weightage to trustworthiness score computed at t0 and t_r−1_. Parameter δ value ranges between [0–1].

#### 3.2.3 Incorporation of belief-of-the majority aspect

An HSP may increase interactions with malicious users to increase their reputation score. HSPs may hire a group of service consumers to give them a high rating for every interaction. Therefore, our trustworthiness assessment scheme focuses on how the majority of service consumers rate a service provider based on the provided service quality rather than concentrating on the frequency of interactions without considering who provided the ratings. The Belief of the majority aspect is added to overcome the value imbalance attack, i.e. higher interactions by a malicious user must not dominate the reputation score calculated for a specific service provider.

In t_r,_, as more time passes, interactions may occur between service consumer *i* and service provider *j*_._ Let *m* represent the count of transactions between service consumer *i* and service provider *j* in *t*_*r*_. Let *R*(*i*,*j*,*C*_*x*_,*t*_*r*_) represent the rating (on a 7-point rating scale) provided by service consumer i (with *PID*_*i*_) to service provider j in time window *t*_*r*_. The *data interpreter service* is a secure mechanism at the RMC that can extract the pseudonymized identifier (*PID*_*i*_) from ${Ticket}_{ijk}^{H}$ to group the ratings provided by a single service consumer in *t*_*r*_ and aggregate them. To incorporate the belief-of-majority aspect, the average rating provided by each service consumer to service provider j at *t*_*r*_ is computed. *R*_*μ*_(*i*,*j*,*C*_*x*_,*t*_*r*_) represents the average of m ratings (based on m interactions) provided by the service consumer i to service provider j in context *C*_*x*_ at *t*_*r*_ (depicted in Eq 3). Let us suppose there are n service consumers who have received service from j in *t*_*r*_. This phase will result in *n* aggregated ratings, *R*_*μ*_(1,*j*,*C*_*x*_,*t*_*r*_)_…….._ *R*_*μ*_(*n*,*j*,*C*_*x*_,*t*_*r*_). This aggregation will result in moving from a discrete domain (to rate the quality of service received from the service provider on a 7-point Likert rating scale) to continuous range.

$$R_{\mu }\left(i,j,C_{x},t_{r}\right)=\frac{\sum_{int_{ij}=1}^{int_{ij}=m}R\left(i,j,C_{x},t_{r}\right)}{m}$$ (3)

#### 3.2.4 Incorporation of the malicious user handling aspect

A Russian mathematician, Pafnuty Lvovich Chebyshev [55][56], established data intervals for any data set, regardless of the shape of the distribution. Chebyshev’s technique can be used to determine what percentage of the data is clustered around the mean even when the data is not normalized. The *median* [55] is a measure of central tendency, which in contrast to the mean, is insensitive to the presence of *outliers*.

The standard deviation (*σ*) is a measure of dispersion that summarizes the amount of dispersion of a set of data values. It specifies how tightly the values in the dataset are clustered around the mean value. If there is high dispersion among the data set [*R*_*μ*_(1,*j*,*C*_*x*_,*t*_*r*_)_…….._ *R*_*μ*_(*n*,*j*,*C*_*x*_,*t*_*r*_)], i.e., the standard deviation of these values $\sigma_{R_{\mu }}(j,C_{x},t_{r})$ is greater than threshold *β* then we apply *Median* ± *dσ* to detect the outliers to reduce the impact of faulty ratings (i.e., given by malicious service consumers) on the overall reputation score of service provider *j*.

CTA protocol computes which rating values among *R*_*μ*_(1,*j*,*C*_*x*_,*t*_*r*_)_…….._ *R*_*μ*_(*n*,*j*,*C*_*x*_,*t*_*r*_) are deviated more than *d* standard deviations from the median reputation score and discards them. Let us suppose there are *n* service consumers in t_r_ who have received services in context *C*_*x*_ from node *j*. After discarding the extreme values among *R*_*μ*_(1,*j*,*C*_*x*_,*t*_*r*_)_…….._ *R*_*μ*_(*n*,*j*,*C*_*x*_,*t*_*r*_), CTA protocol retains the ratings of the p service consumers, i.e., *R*_*μ*_(1,*j*,*C*_*x*_,*t*_*r*_)_…….._ *R*_*μ*_(*p*,*j*,*C*_*x*_,*t*_*r*_).

At the time of reputation calculation, based on the provided trustworthiness value of a trustee (in a similar context), the credibility of the service consumer as a recommender is updated by the CTA. The credibility of the service consumer as a recommender lies between 0 and 1. A default value of “0.5” is assigned to the credibility of a service consumer as a recommender [13][14]. The credibility of each service consumer *i* who provided *R*_*μ*_(*i*,*j*,*C*_*x*_,*t*_*r*_) is among the discarded values and will be decreased by threshold *θ*_1_.

To compute the credibility of the remaining service consumers who provided ratings in context *C*_*x*_ in *t*_*r*_, we compute the relationship of each rating (in a group of *R*_*μ*_(1,*j*,*C*_*x*_,*t*_*r*_)_…….._ *R*_*μ*_(*p*,*j*,*C*_*x*_,*t*_*r*_)) to the mean $\mu_{R_{\mu }}(j,C_{x},t_{r})$. The Z-score [55] tells us how far rating *R*_*μ*_(*k*,*j*,*C*_*x*_,*t*_*r*_) is from $\mu_{R_{\mu }}(j,C_{x},t_{r})$ (depicted in Eq (4)) in terms of standard deviation $\sigma_{R_{\mu }}(j,C_{x},t_{r})$ (depicted in Eq (5)) and where it is relatively positioned in the distribution. Z-scores [55] allow an accurate comparison of ratings scores to one another and placement on a normal distribution curve. For the Z-score, the measure of dispersion and the measure of central tendency are used together. The Z-score will be large for a value that is highly deviated from the mean. In the case of distribution with tightly clustered data values around the mean (i.e., small standard deviations), a very small deviation from the mean gives an approximately similar Z-score.

$$\mu_{R_{\mu }}\left(j,C_{x},t_{r}\right)=\frac{\sum_{i=1}^{i=p}R_{\mu }\left(i,j,C_{x},t_{r}\right)}{p}$$ (4)

$$\sigma_{R_{\mu }}\left(j,C_{x},t_{r}\right)=\sqrt{\frac{\sum_{i=1}^{i=p}{\left(R_{\mu }\left(i,j,C_{x},t_{r}\right)-\mu_{R_{\mu }}\left(j,C_{x},t_{r}\right)\right)}^{2}}{p}}$$ (5)

The Z-score of each value among (*R*_*μ*_(1,*j*,*C*_*x*_,*t*_*r*_)_…….._ *R*_*μ*_(*p*,*j*,*C*_*x*_,*t*_*r*_)) is computed. The credibility *Cr*_*k*_ of each service consumer *i* whose provided rating (*R*_*μ*_(*i*,*j*,*C*_*x*_,*t*_*r*_)) Z-score value lies in the range [> = -1, < = +1] will increase by threshold *θ*_2_. The credibility of each service consumer *i* whose provided rating *R*_*μ*_(*i*,*j*,*C*_*x*_,*t*_*r*_) Z-score value lies in the range [<-1 AND > = -2, >+1 AND < = +2] will decrease by threshold *θ*_3_, where threshold *θ*_1_ > *θ*_3_. The credibility of each service consumer *i* (where i ranges between 1. p)) whose provided rating *R*_*μ*_(i,*j*,*C*_*x*_,*t*_*r*_) Z-score value lies in the range [< = -2, or > = +2] will decrease by threshold *θ*_1_ and all such values will be discarded. After discarding all such ratings among *p* ratings, we are left with *O* ratings (*R*_*μ*_(1,*j*,*C*_*x*_,*t*_*r*_)_…….._ *R*_*μ*_(*O*,*j*,*C*_*x*_,*t*_*r*_)).

The trustworthiness score $T(j,C_{x},t_{r})$ (depicted in Eq (6)) of service provider j at time *t*_*r*_ is then calculated as the weighted average.

$$T\left(j,C_{x},t_{r}\right)=\frac{\sum_{i=1i\neq j}^{O}R_{\mu }\left(i,j,C_{x},t_{r}\right)*Cr_{k}}{\sum_{i=1,i\neq j}^{O}Cr_{k}}$$ (6)

$$T_{RMC}\left(j,C_{x},t\right)=\alpha *T\left(j,C_{x},t_{0}\right)+\left(1-\alpha \right)*T\left(j,C_{x},t_{r}\right)$$ (7)

After the passage of t, time window *t*_*r*_ will expire and the trustworthiness (reputation) of service provider j at t will be calculated using Eq 7. *α* is a weighting factor that ranges between [0–1] and is used to weight past and recent trustworthiness (reputation). Assigning lower weights to *α* means that low weight is given to the trustworthiness value calculated during *t*_0_. If the $T(j,C_{x},t_{r})$ is calculated for the first time, then a default reputation value will be considered for $T(j,C_{x},t_{0})$. At the second and forthcoming re-evaluations of the trustworthiness score of service provider j upon the expiration of the evaluation period, before calculating the updated value of $T(j,C_{x},t_{r})$, the current value of $T(j,C_{x},t_{r})$ will be copied to $T(j,C_{x},t_{r-1})$ to update $T(j,C_{x},t_{0})$ using Eq 8. *β* is a weighting factor that ranges between [0–1].

$$T\left(j,C_{x},t_{0}\right)=\beta *T\left(j,C_{x},t_{0}\right)+\left(1-\beta \right)*T\left(j,C_{x},t_{r-1}\right)$$ (8)

The overall reputation of service provider j at *t* will be calculated by the RMC using Eq (7). The $T_{RMC}(j,C_{x},t)$ value will be entered into Eq (1) along with other required values to obtain $FT(j,C_{x},t)$.

## 4. Formal verification of the anonymity scheme

### 4.1 High-Level Petri Nets (HLPN)

Petri nets as a powerful mathematical and graphical notation for modelling can be applied to many systems characterized as being concurrent, asynchronous, distributed, parallel, non-deterministic, or stochastic [57]. In this paper we have used High-Level Petri Nets (HLPN). (Readers are encouraged to see [57] for more details).

### 4.2 SMT-Lib and Z3 solver

Satisfiability Modulo Theories (SMT) is an area of automatic deduction responsible for checking the satisfiability of formulas over some logical theories of interest. SMT has the roots from Boolean Satisfiability Solvers (SAT) [58]. SMT is commonly used for deductive software verification. Moreover, SMT is also used for planning, model checking, and automated test generation finding in the are of computer science [59]. SMT-LIB is supported by multiple solvers including Boolector, MathSAT5, Z3, and OpenSMT [60]. Z3 [61] solver -a high performance theorem prover developed at Microsoft Research- is used in our study.

### 4.3 Formal modelling of GID_i_ generation algorithm

**Fig 4:**
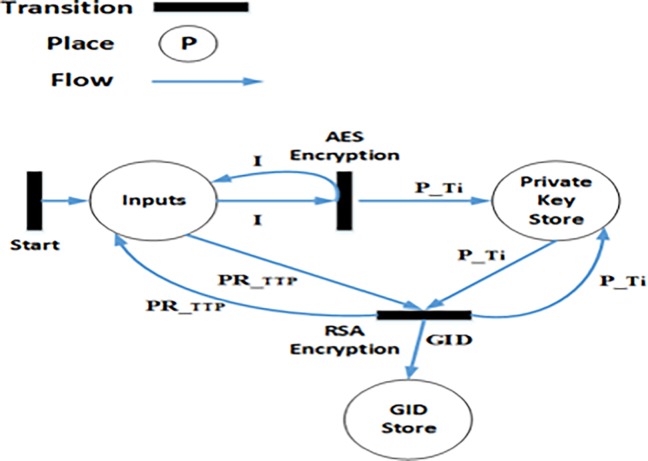
Algorithm 1: Generation of GID (Petrinet).

The GID_i_ generation process is described in Algorithm 1 (Fig 1). The HLPN model of Algorithm 1 is depicted in Fig 4. Firstly, places, data types, and respective mappings are identified for modelling. The places and the data types that are mapped to these places are given in Table 1. The *Start* transition allows new tokens to be entered in the HLPN model. In the first step towards GID_i_ creation, the *AES Encryption* transition (using the rules shown in (9)) generates the $P_{i}^{T}$ by encrypting *NHS*_*NO*_*i*_ using ${SecK}_{TTP}^{T}$. The TTP then uses its private key (${PRI}_{TTP}^{T}$) to encrypt $P_{i}^{T}$ to finally generate GID_i_, as shown in (10).

**Table 1:** *GID*_*i*_ generation mappings.

Places	Mappings
φ (Inputs)	*ℙ* (${SecK}_{TTP}^{T}$ × *NHS*_*NO*_*i*_ × ${PR}_{TTP}^{T}\times Nonce$)
φ (Private Key Store)	*ℙ (P*^*T*^_*i*_*)*
φ (Global ID Store)	ℙ (GID)

$$\begin{array}{c} R\left(AES-Encryption\right)=\forall \;i\in I,\forall \;p\_\mathrm{ti}\in P\_\mathrm{TI}|\\ \;p\_\mathrm{ti}≔\mathrm{AES}-\mathrm{Encryption}\;\left(i\left[1\right],i\left[2\right]\right)\wedge \\ \;P\_{\mathrm{Ti}}^{\prime }=P\_\mathrm{Ti}\cup \left\{p\_\mathrm{ti}\right\} \end{array}$$ (9)

$$\begin{array}{c} R(GID\;RSA\;Encryption)=\forall \;pr\_ttp\in PR\_TTP,\forall \;p\_ti\in P\_TI,\forall \;gid\in GID|\;gid\\ \;≔GID\;RSA\;Encryption\;(pr\_ttp[1],pr\_ttp[2],pr\_ttp[3])\wedge GID\prime =GID\cup \left\{gid\right\} \end{array}$$ (10)

### 4.4 Formal modelling of LID_ij_ generation algorithm

**Fig 5:**
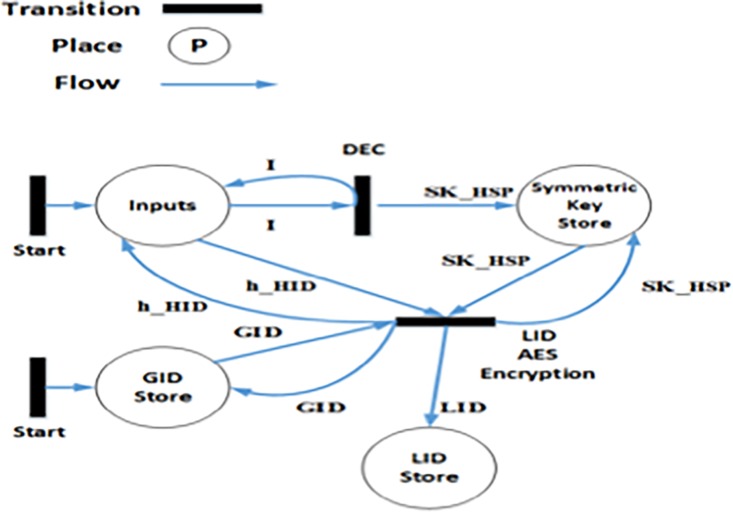
Algorithm 2: Generation of LID (Petrinet).

The Algorithm 2 (Fig 2) discussed in Section 3.1.2 illustrates the steps for creating *LID*_*ij*_ for the patient. The HLPN model and the mappings of data types to the places are shown in Fig 5 and Table 2, respectively. As given in (11), the HSP use ${SecK}_{j}^{HSP}$ for decrypting ${E\_SK}_{j}^{HSP}$. The *LID*_*ij*_ is generated by encrypting ***h***(*HID*_*j*_) and *GID*_*i*_ using ${SK}_{j}^{HSP}$, as shown in (12). Hospital ID (*HID*_*j*_) is generated for each hospital by the HCA.

**Table 2:** *LID*_*ij*_ generation mappings.

Places	Mappings
φ (Inputs)	ℙ (${SecK}_{j}^{HSP}$ × ${E\_SK}_{j}^{HSP}$ × h(*HID*_*j*_)x nonce)
φ (Symmetric Key Store)	ℙ (SK^HSP^)
φ (GID Store)	ℙ (GID)
φ (LID Store)	ℙ (LID)

$$\begin{array}{c} R\left(DEC\right)=\forall \;i\in I,\forall \;\mathrm{sk}\_\mathrm{hsp}\in SK\_HSP\;|\\ \mathrm{sk}\_\mathrm{hsp}:=\mathrm{DEC}\left(i\left[1\right],i\left[2\right]\right)\wedge \\ SK\_HSP^{\prime }=SK\_HSP\cup \left\{\mathrm{sk}\_\mathrm{hsp}\right\} \end{array}$$ (11)

$$\begin{array}{c} R\left(LID\;AES\;Encryption\right)=\forall \;h_{\_\mathrm{hid}}\in H\_HID,\forall \;\mathrm{sk}\_\mathrm{hsp}\in SK\_HSP,\forall \;\mathrm{gid}\in GID,\forall \;\mathrm{lid}\in LID|\\ \mathrm{lid}:=\mathrm{LID}\;\left(gid\left[1\right],gid\left[2\right],gid\left[3],gid[4\right]\right)\wedge \\ LID^{\prime }=LID\cup \left\{gid\left[1\right],gid\left[2\right],gid\left[3],gid[4\right]\right\} \end{array}$$ (12)

### 4.5 Formal modelling of ticket-generation algorithm

**Fig 6:**
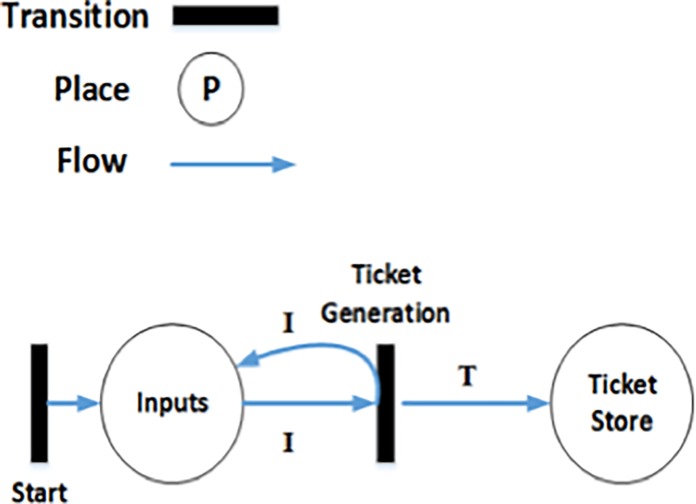
Algorithm 3: Generation of ticket (petrinet).

In Algorithm 3 (Fig 3), unique tickets are generated for every patient, as discussed in Section 3.1.3. The HLPN model and the mappings of places to the data types are represented in Fig 6 and Table 3, respectively. The unique ticket number *Ticket*_*ijk*_ is generated by the ticket-generation component at the HCO j for patient *i* based on the information including service consumer *GID*_*i*_, *PRPID*_*j*_^*HSP*^, ContextID_***c***_, date and time. The same ticket can be used for the verification of the algorithm, as in (13).

**Table 3:** *Ticket*_*ijk*_ generation mappings.

Places	Mappings
φ (Inputs)	ℙ (GID_j_× ContextID_***c***_× *PRPID*_*j*_^*HSP*^ ×date × time× nonce)
φ (TicketStore)	ℙ (Ticket)

$$\begin{array}{c} R\left(Ticket\;Generation\;AES\;Encryption\right)=\forall \;i\in I,\forall \;t\in T\;|\\ t≔\mathrm{Ticket}\;\mathrm{Generation}\;\left(i\left[1\right],i\left[2\right],i\left[3\right],i\left[4\right]\right)\wedge \\ T\prime =T\cup \left\{i[1],i[2],i[3],i[4],i[5]\right\} \end{array}$$ (13)

### 4.6 Z3 solver for verification

**Fig 7:**
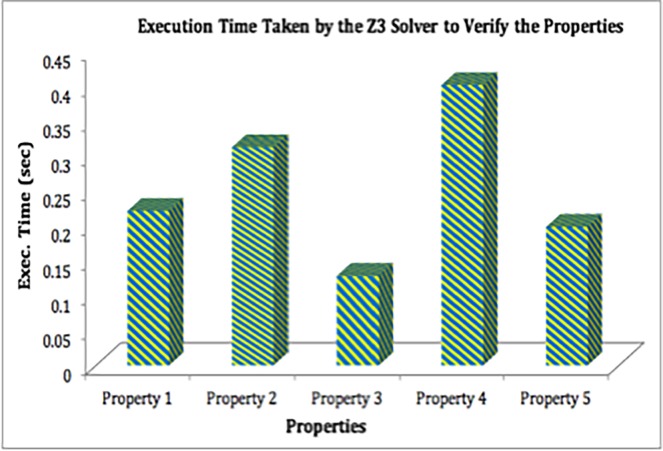
Execution time taken by the Z3 solver to verify the properties.

After verification Z3 solver categorizes results as satisfiable (sat) or un-satisfiable (unsat). The solver tries to depict the violation of the formula *f* by generating a counter example, in case the result is *sat* [61]. However, if the result is unsat, then formula *f* holds in *M* up to the bound *k* (in our case *k* is exec. time). Fig 7, depicts the computation time taken by the Z3 solver for verification of the following properties.

Property 1: This property verifies that *NHS*_*No*_*i*_ of a patient i is used to generate a unique *GID*_*i*_.

Property 2: This property verifies the uniqueness and correctness of the generated *GID*_*i*_ for each patient.

Property 3: This property verifies the uniqueness and correctness of the generated *LID*_*ij*_ for patient i.

Property 4: This property verifies whether the generated *LID*_*i*_ is correct or not.

Property 5: In Property 5 it is verified whether the Ticket is generated correctly or not.

## 5 Results and discussion

### 5.1 Security analysis

#### 5.1.1 Threat model

It is assumed that, in contrast to internal attackers, all external attackers are unauthenticated and unauthorized entities. Attacks such as DoS attacks, eaves dropping and traffic jamming etc., are beyond the scope of this paper. For simplicity, in our model, each service consumer and service provider is assigned a unique anonymous local ID at each HCO and a unique global identity that maps all local IDs to a single ID. An entity is allowed not to provide any real identity information. We assume that there is no correlation between service provider popularity in the system and its likelihood to be a target. Instead, each service provider has an equal probability of being targeted. An existing service consumer can leave his/her account and register himself/herself as a new user. A misbehaving service consumer may provide a false recommendation randomly, with a certain probability or for certain interactions, to increase or decrease the reputation of the service provider. Groups of entities may attack a service provider by giving out unfair ratings and recommendations. An adversary as a service provider may provide false information by lying about their reputation level or exploit to gain an unfair reputation.

The entities (the HCA, TTP, HCO, and the RMC) are considered trustworthy that have been authenticated and authorized by the system. An adversary can compromise the TTP, HCO, or RMC, but not all three. Therefore, the entities cannot conspire. By assuming that all security-enhancing functionalities employed in the system are well-deployed, all secret keys are stored and physically secure. The server is considered trustworthy in terms of the different functions it can perform such as trust evaluation and reputation management. Communication occurs through a secure communication channel. Patient records have been de-identified in advance. In our scheme, all the attributes that can identify a patient are with HCA.

#### 5.1.2 Defence model

This section presents the defense model of the proposed scheme.

**5.1.2.1 Building up reputation under multiple pseudonyms**. In Sybil attack, a malicious user registers himself/herself using several forged identities in the TRS with the intention of acting maliciously on behalf of the forged entities. Our system does not allow a malicious service consumer to acquire multiple identities to deliberately hurt the reputation of a single HSP or group of HSPs. The service consumer is assigned a unique pseudonym to rate each interaction. Our system assigns each user *i* a unique *GID*_*i*_ based on his NHS number. Moreover, based on the *GID*_*i*_ a unique *LID*_*ij*_ is generated at each HCO *j*. Furthermore, based on *LID*_*ij*_ a unique pseudonymized identifier is generated to rate the quality of service provided by the service provider. If the service consumer leaves the system and then re-enters the system, it will not impact his NHS number. Similarly, each time when the service consumer will request for new *GID*_*i*_, based on the NHS number the generated *GID*_*i*_ will be similar to the one as issued earlier.

**5.1.2.2 TTP tries to compromise user’s privacy**. Consider the scenario that the TTP is compromised, and being controlled by an adversary. TTP does not store any information about the users, instead use the pseudonym function that supports reversibility (conversion of *GID*_*i*_ to *NHS*_*No*_*i*_ and vice versa). To request for the reversibility, the adversary must be an authorized user. Moreover, even if the adversary is an internal attacker, he/she cannot decrypt *GID*_*i*_ to get *NHS*_*No*_*i*_ of service consumer *i*, unless he/she also acquire secret keys and necessary information about the reversible pseudonym function. Moreover, even if the adversary gets successful in knowing about the *NHS*_*No*_*i*_, to collect the patient identification information from the HCA, the adversary must be authorized user.

**5.1.2.3 RMC tries to compromise user privacy**. An internal user might try to collect personal information about service consumer by linking up all the records of a service consumer to find out which doctor’s the service consumer has visited and in which context. Therefore, in our proposed scheme personal identification information of a service consumer is de-identified. Moreover, each HCO j assign different anonymous identity to the service consumer *i* to provide rating to the service provider in *j*. Furthermore, ticket generated for each interaction is unique, therefore, an adversary cannot link the records of a service consumer. Unique identifier is used in place of Date and Time so that an internal adversary should not be able to guess service consumer based on the date and time on which he/she received the service.

**5.1.2.4 Estimating attacks**. In our scheme, probability of guessing attacks is low as patient pseudonyms (*GID*_*i*_, *LID*_*ij*_, and *Ticket*_*ijk*_) are not generated by using modest methods.

**5.1.2.5 Context/criteria compatibility.** Our proposed scheme calculates reputation scores of service provider relevant to each context rather than aggregating ratings provided by service consumers to service providers in different contexts. Consider example scenario, all surgeons are physicians, but not all physicians are surgeons. A negative rating of a doctor as a physician should not influence his/her reputation score of acting as a surgeon. In our proposed scheme, using ContextID_***c***_ can attain unlink ability of a service consumer actions in different contexts.

**5.1.2.6 Reducing the influence of unfair ratings.** Bad mouthing attack occurs when a dishonest service consumer tries to hurt the reputation of one or more good HSPs by assigning unfairly low ratings to them. Where as in ballot stuffing attack, HSPs engage in many fake dealings to artificially inflate their reputations and ratings. In each *t*_*r*_, to reduce the impact of malicious service consumers on the overall reputation of the service provider, three initiatives are taken: (i) belief of the majority aspect is incorporated, (ii) to overcome the problem of hurting the reputation of good service providers, ratings that are deviated more than d standard deviation from the median are discarded (using *Median* ± *dσ* and Z-score method), (iii) to discourage malicious activities and to reduce the impact of adaptive ratings the credibility of service consumer is computed.

Moreover, for every interaction between a service provider and a service consumer the ticket generated by HCO is unique and time-bounded, by using which service consumer rate the service quality. Ticket expiry helps to address the situation where the HSP restricts the service consumers to provide ratings until and unless advised by HSP (i.e., HSP wants to use ratings for such interactions when HSP thinks that his/her reputation score will fall in the current *t*_*r*_ because of providing low quality service to the service consumers). Furthermore, the HSP may hire a group of service consumers, collect the tickets from them and use them (self-promotion attack). To overcome such attack, the ticket is provided to the RMC by HCO as part of attribute certificate.

**5.1.2.7 Grudge attack.** Using cryptographic technique, the ticket is generated for every interaction between service consumer and service provider to allow service consumers to rate anonymously. The ticket will allow them to rate fearlessly, without expecting any kind of retaliation from service providers and without the fear that provided anonymous ratings by one service consumer can be linked to each other by the adversary to build a profile of that individual or to find the similarity between the service consumers. The encrypted tuple values in the RMC cannot be exploited without the private key information. Moreover, information contained in encrypted tuple values cannot be inferred by simply comparing encrypted values.

**5.1.2.8 Time sensitivity of reputation.** Each service consumer is given a unique one-time ticket ID after every interaction that is time bounded. A service consumer can only use this ticket with in the limited validity period after which this ticket get expired and cannot be used. To avoid traitor attack, where dishonest service consumers establish trust by fair interactions in the start and later misuse this trust by behaving maliciously. Our scheme divides the overall ratings for each HSP in two time windows *t*_0_ and *t*_*r*_. We use the two parameters *α* and *δ*, to incorporate the time-varying aspect. The HCA also independently calculates the reputation score of the service provider using the information such as work experience, education, complaints, praises, promotions etc.

**5.1.2.9 Value imbalance & traitor attack.** The HSPs may engage in many fake dealings to artificially inflate their reputations. Therefore, our scheme focuses on how the majority of the service consumers rate the service provider. Let us consider an example scenario, in *t*_*r*_, 20 users interacted with the service provider. Out of which, 15 interacted only once, whereas the rest of 5 interacted 30 times each to perform ballot stuffing (i.e., to increase the trustworthiness score of the service provider). In such a scenario, if we calculate the reputation based on the number of interactions then in that case the reputation score for the service provider will not accurately represent the views of all service consumers, as the five users who interacted more will dominate the reputation score. The scheme proposed is efficient enough to handle such attacks. Our scheme allows the service consumer to rate considering 7-point rating scale instead of rating the HSP as true or false.

### 5.2 Experimental evaluation

We have performed experiments to compute the effect of ballot stuffing and bad mouthing attacks on the reputation score of the service providers. The proposed scheme is evaluated using Lenovo ThinkPad running on Windows 10 with 2.30 GHz Intel Core i5. The software used is Visual Studio Ultimate 2012 with MYSQL database. Results are shown in graphical form i.e. on x-axis system has number of attackers while on y–axis accuracy of our proposed system.

We set the value of d as 1.2 in Median ∓ d σ inequality in all of our experiments and dispersion threshold β is set to value >1.3. In both set of experiments 100 patients perform number of interactions between [1,5] with the service provider j in the similar context. Values of credibility parameters are set as follows: (i) parameter *θ*_1_ is set as 20%, (ii) *θ*_2_ is 10%, and (iii) *θ*_3_ is set as 15%. Initial credibility of service consumer as recommender is set as 0.5. To compute the accuracy of the reputation score, we compared the reputation score computed through our scheme with the average of reputation values provided by non-malicious users and with the scheme presented in [39].

### 5.2.1 Experiment 1: Bad mouthing attack: Impact on reputation accuracy

**Fig 8:**
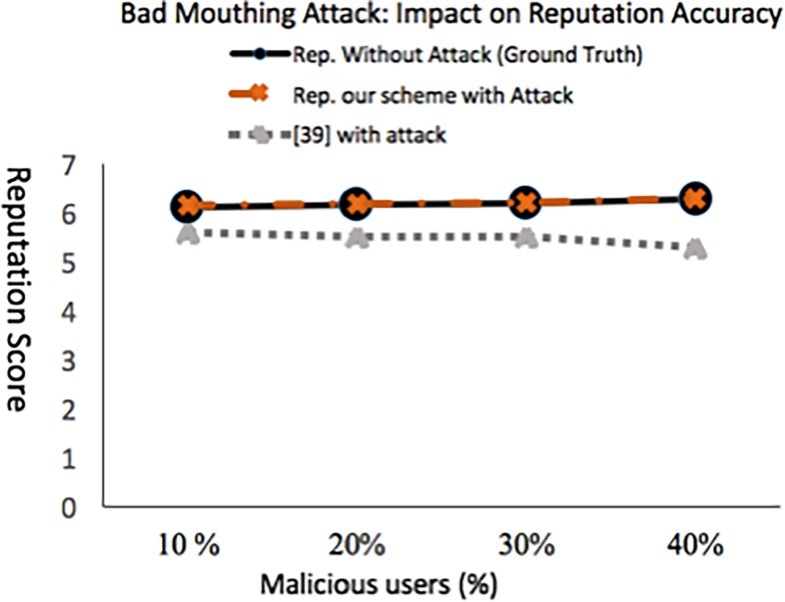
Impact of bad mouthing attack on the accuracy of reputation score when attack is done in *t*_*r*_.

Accuracy of our scheme in terms of accurately computing reputation score as the percentage of malicious user’s increases in *t*_*r*_ is shown in Fig 8. Like [39], the malicious users are the ones whose aggregated rating lies in the range [1,2] where as the non-malicious service consumers aggregated rating lies in the range [4,7]. We made the adversaries to be colluding and attack a single service provider to gain more support from each other.

To effectively measure the accuracy of detection of dishonest recommendations, we use Matthews Correlation Coefficient (MCC) [55][39]. MCC is a measure of the quality of binary (two-class) classifications taking in to considerations true and false positives and negatives. True Positives (TPs) represents the number of malicious service consumers that are truly detected as malicious. The number of service consumers that are not malicious but are detected as malicious, denoted as False Positives (FPs). The number of malicious service consumers that are not detected as malicious, denoted as False Negatives (FNs). True Negatives (TNs) represents the number of non-malicious service consumers that are truly detected as non-malicious service consumers.

$$MCC=\frac{\left(TP*TN)-(FP*FN\right)}{\sqrt{\left(TP+FP)(TP+FN)(TN+FP)(TN+FN\right)}}$$

**Fig 9:**
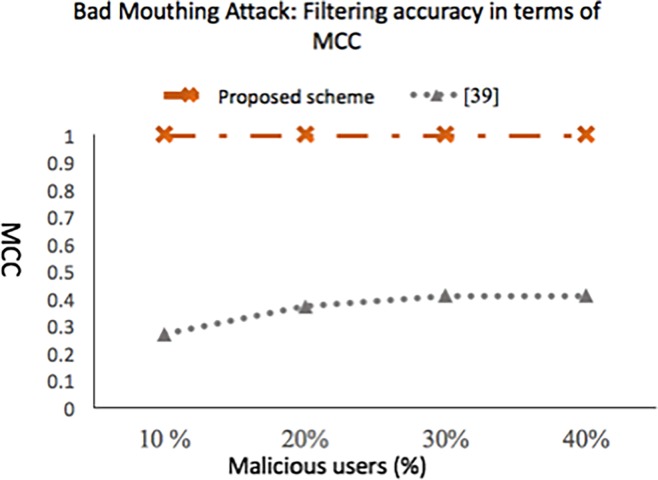
Filtering accuracy in terms of MCC (bad mouthing attack done in *t*_*r*_).

Fig 8 shows the impact on a service provider reputation with increase in percentage of malicious users. Using [39] the reputation inaccuracy increases with increase in the number of malicious users. In contrast, with our scheme the reputation score of a service provider is not impacted much with the increase in malicious users. As shown in Fig 9, the proposed approach efficiently detects dishonest recommendations. This is evident from a constant MCC of +1 with the increase in dishonest recommenders percentage from 10% to 40%. Our scheme behaves poorly in the case where the number of dishonest recommenders increases above 40%.

The scheme presented in [39] do detect the malicious users accurately but is suffered from false positives as shown in Fig 9. When the number of dishonest recommenders is 10%, the number of honest recommenders who rated service provider in the range [4.1, 6] are correctly detected as non-malicious, whereas those who rated the service provider in highest category (i.e., between [6.1,7]) are falsely detected as malicious (the number of recommenders who rated service provider in highest category are 50). Therefore, the MCC value is lower, when the dishonest recommenders are 10%. With the increase in the percentage of dishonest recommender’s the MCC increases slightly. The reason behind is number of TPs (dishonest recommenders that are correctly detected as malicious by the scheme) increases where as number of FPs approximately remains the same (honest recommenders who rated between [6.1, 7] falsely detected as malicious by the scheme are 45 when malicious recommenders are 20%, 42 at 30%, and 40 at 40%).

### 5.2.2 Experiment 2: Ballot-stuffing attack- impact on reputation accuracy

**Fig 10:**
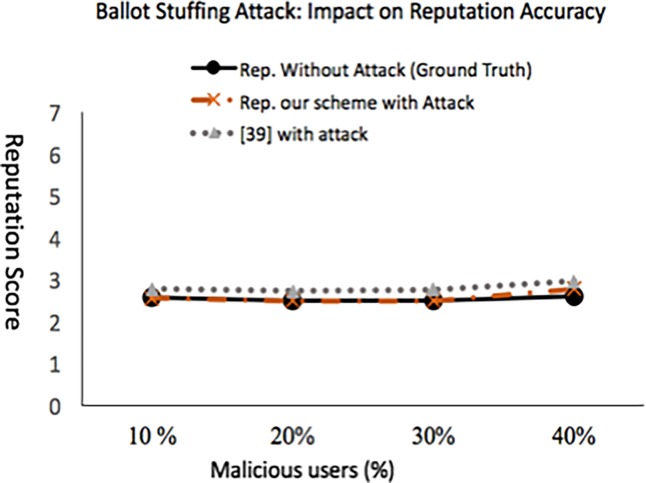
Impact of ballot-stuffing attack on the accuracy of reputation score when attack is done in *t*_*r*_.

**Fig 11:**
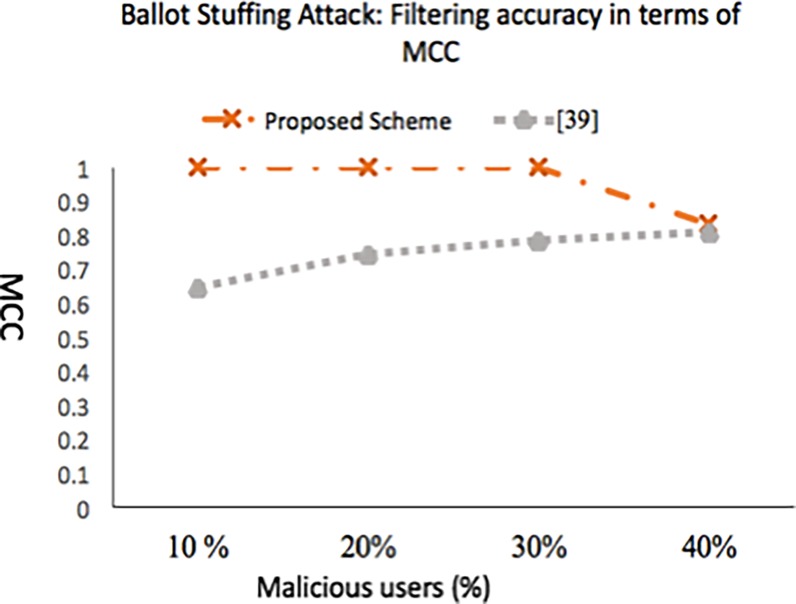
Filtering accuracy in terms of MCC (ballot-stuffing attack done in *t*_*r*_).

Accuracy of our scheme in terms of accurately computing reputation score as the percentage of malicious user’s increases in *t*_*r*_ is shown in Fig 10. Like [39], the malicious users are the ones whose aggregated rating (incorporation of belief of majority aspect) lies in the range [6, 7] where as the non-malicious (honest) service consumers aggregated rating lies in the range [1, 4]. We made the adversaries to be colluding and attack a single service provider to gain more support from each other. Our scheme allows to detect all malicious users, but increase in the percentage of malicious users above 30% resulted in detection of false positives (service consumers that are not malicious but which rated the service provider very low are added to malicious category, denoted as FP), which impacted the final computed average score. The accuracy of the reputation score is not adversely impacted as long as the adversaries are less than the good participants in the network. Fig 11 depicts that even in the presence of 40% malicious users the MCC is not dropped much showing the survivability of our approach. However, the rapid increase in false positive ratio and false negative ratio shows that the model is not effective when the number of dishonest recommenders exceeds 40%.

The scheme presented in [39] do detect the malicious users accurately but is suffered from false positives as shown in Fig 11. When the number of dishonest recommenders is 10%, the number of honest recommenders who rated service provider in the range [1.1, 4] are correctly detected as non-malicious, whereas those who rated the service provider in lowest category (i.e., between [0,1]) are falsely detected as malicious (the number of recommenders who rated service provider in lowest category are 11). Therefore, the MCC value is lower, when the dishonest recommenders are 10%. In Fig 11, the MCC increases slightly with the increase in percentage of dishonest recommender’s. The reason behind is that the false positives remains the same (the number of recommenders who rated service provider between [0,1] are 11), where as the number of TPs (dishonest recommenders that are correctly detected as malicious by the scheme) increases.

## 6. Conclusion & future work

One of the important requirements of TRSs in the health sector is rating secrecy, which mandates that the identification information about the service consumer should be kept secret to prevent any privacy violation. The work presents a framework that solves the problem of reconciling trust with anonymity in the health sector. Our solution includes *Anonymous Reputation Management* (ARM) and context-aware trust assessment protocols. The main contributions of the ARM protocol include (i) avoiding linking anonymous ratings provided by a service consumer, (ii) keeping the identities of service consumers concealed from service providers, and (iii) unveiling service consumer’s and service provider’s identities if required by the system. In this work, to rate quality of service provided by service provider a unique time-bound ticket is generated by health care organization. A context-aware trust assessment scheme is presented which compute the reputation of service providers. Our simulation results verify the accuracy of the proposed context-aware trust assessment scheme. Moreover, through formal verification, we verify the anonymous reputation management protocol.

There are still a lot of open research issues that needs to be addressed related to designing and developing a context-aware TRS including reliability, privacy, security, and robust computational models, among others. Generic and comprehensive trust model is required that can establish context-aware trust considering all the dimensions of e-health, namely, trustee (HSP and HCO), trustor, information content, and information technology. Information Technology dimension of trust consider the attributes that deals with the safe and effective exchange of data. The information content dimension of trust refers to attributes that determine the trustworthiness of data such as accuracy, completeness, timeliness, relevance, legibility, accessibility, usefulness and confidentiality.

## Supporting information

S1 Table Badmouthing experiment datasheet. (XLSX)

S2 Table Ballot-stuffing experiment datasheet. (XLSX)
